# Segmental acquisition of Brazilian Portuguese: onset simple, complex and coda

**DOI:** 10.1590/2317-1782/20212020439

**Published:** 2022-01-12

**Authors:** Marizete Ilha Ceron, Simone Nicolini de Simoni, Gabriel Agustín Urrutia Urrutia, Márcia Keske-Soares

**Affiliations:** 1 Prefeitura de Santa Cruz do Sul - Santa Cruz do Sul (RS), Brasil.; 2 Programa de Pós-graduação em Distúrbios da Comunicação Humana, Universidade Federal de Santa Maria – UFSM - Santa Maria (RS), Brasil.; 3 Programa de Pós-graduação em Distúrbios da Comunicação Humana, Universidade Federal de Santa Maria – UFSM - Santa Maria (RS), Brasil.; 4 Universidad de Talca - Talca, Region del Maule, Chile.; 5 Laboratório de Fala – LabFala, Departamento de Fonoaudiologia, Universidade Federal de Santa Maria – UFSM - Santa Maria (RS), Brasil.

**Keywords:** Speech, Phonological Acquisition, Children Language, Children, Phonology

## Abstract

**Purpose:**

To present and analyze the acquisition segmental curve of Brazilian Portuguese in simple and complex onset position and coda position.

**Methods:**

857 children with typical phonological acquisition participated in it, aged between 3:0 and 8:11, divided into age groups every 6 months. Participants were assessed using INFONO phonological assessment software. After analyzing the results, acquisition curves were drawn up for the segments analyzed in the different structures (simple and complex onset position and coda position).

**Results:**

It was noted that, in simple onset position, some segments were acquired before 3:0 (stops, nasal and fricative, /f, v, s, z/. The /ʃ/ and /l/ segments were acquired at 3:0, /ʒ/ and /x/ at 3:6, /ʎ/ at 4:0, /r/ at 4:6; in coda position /N/ and /L/ were acquired before of 3:0, /S/ at 3:6 and /r/ at 4:6; in complex onset position, the structures composed by Fricative + /r/ and Stop + /r/ were acquired at 5:0, Stop + /l/ at 5:6 and Fricative + /l/ at 6:0.

**Conclusion:**

Analyzing the acquisition curve is essential, as it provides a reference on the age of acquisition of segments in different syllabic structures. The acquisition curve contributes to the early identification of delays in phonological acquisition process enabling a timely referral for speech therapy intervention.

## INTRODUCTION

The phonological acquisition of Brazilian Portuguese (BP) is a relevant topic to be researched and discussed in scientific community. This knowledge provides an understanding of the acquisition process of the language segments and how it happens for different children^(1)^.

The BP consonant inventory consists of 19 segments, from the phonological point of view it is distributed in sound class, forming the stops (/p, b, t, d, k, g/), nasals (/m, n, ɲ), fricatives (/f, v, s, z, ʃ, ʒ/), liquids (/x, ʎ, l/) e the tap /ɾ/. These segments are further divided according to the syllabic structure of the language into simple, complex and coda onset position^(2)^.

The phonological acquisition of BP consonants begins gradually and progressively. It is estimated that at approximately 5 years old, the child presents the complete phonological inventory or close to that of the adult. The order of acquisition and development of the sound classes is stops and nasals, followed by fricatives and liquids including tap /ɾ/, this last class is considered later domain^(3,4)^. This same acquisition order is also referred to in international studies^(5,6)^.

The complex onset is the later acquisition structure, as it presents greater production complexity^(7)^. A study^(8)^ showed that there is no difference in age of acquisition between structures composed of obstruent+/ɾ/ and obstruent+/l/ and that the complex onset was acquired between 4:0 and 4:6.

It is important to know and understand the typical phonological acquisition process in order to be able to identify, diagnose and treat children with suspected alterations in this acquisition chronology. In the literature^(9-15)^ there are differences in ages related to the acquisition of segments in both the simple onset and complex onset structures. There is a greater discrepancy in the findings related to complex onset. These differences in the age of acquisition may be due to:

- Methodological variations of the researches, for example, the use of different age groups, some use 12 months^(10,11)^ others 6 months^(3,8)^, another 4 months^(14)^, another is longitudinal^(2,13)^. Phonological acquisition is gradual and fast in the first years of life, so analyzing very large ranges may not show the real age of acquisition and domain of a particular phoneme;
- Forms of data collection, for example, some studies^(10,11)^ created specific lists that were elicited by naming isolated pictures, another^(8)^ with naming pictures of a non-standardized phonological instrument, still others^(3,14)^ by naming pictures from a standardized phonological instrument (instrument that was developed following psychometric criteria to be used) and another^(2)^ by spontaneous production with the aid of objects. The importance of using standardized instruments in the assessment process is increasingly known and that these instruments present psychometric criteria of validity and reliability for the reliability of the assessment results^(16,17)^. Thus, the form of data collection is an important factor for studies to be compared;
- Reduced samples. The sample size in each age group is important so that the information can be generalized to a population. Some national studies were carried out with an extremely small number of children^(2,8,13)^ while others with slightly more expressive samples^(10,11)^. It is believed that the phonological assessment performed directly interferes with the sample as it is an assessment whose analysis is time-consuming, demanding of time, which makes it difficult to carry out large quantities. However, the sample size is a factor highlighted in the literature^(17,18)^ in the sense that the groups of participants must be large enough to guarantee comparisons of subgroups with the scores of the general population. International studies that analyze acquisition mention superior samples^(5,6)^;
- Research carried out in different regions of Brazil (language heterogeneity). The language is the same, but with peculiar features of each region, the age of acquisition and domain of some phonemes may vary a little.

Thus, it is believed that these differences can bring about important divergences in the ages of acquisition and domain of the BP segments. This makes it essential to carry out more studies on the phonological acquisition of BP with younger age groups, with standardized instruments for the population, with a greater number of subjects per age group and to keep the data up to date, as the language is dynamic and is constantly changing.

It is expected that the acquisition curve of segments performed from phonological acquisition data of typical children can determine whether a child's speech development progresses normally or is delayed in this acquisition process and, thus, assist in the diagnosis and intervention before of the literacy period.

Given the above, this work aimed to present and analyze the segmental acquisition curve of BP in simple and complex onset position and coda position. For that, there are the following hypotheses: the phonological acquisition occurs in a dynamic and gradual way, starting with the less complex segments until reaching the more complex ones; a segment begins to be produced (age of production), goes on to acquisition (age of acquisition) and finally reaches complete domain of speech (age of domain); that the age of production, acquisition and domain of the segments are different.

## METHODS

This study is descriptive, cross-sectional and quantitative. The project was approved by the Research Ethics Committee under number 23081.005433/2011-65. All children were authorized by their parents and/or guardians to participate in the research by signing the Informed Consent Form. The Research and Health Guidelines were complied with, in accordance with Resolution 466/2012.

### Participants

The sample was of convenience. Twelve schools from two cities in Rio Grande do Sul (Brazil) were selected (8 public and 4 private), a total of 1806 children were invited to participate in the study, of which 1274 (70%) were authorized by their parents to participate. All children were aged between 3 years old and 8 years and 11 months old.

Eligibility criteria were: monolingual children from BP with typical phonological acquisition, with no history or suspicion of hearing and neurological alterations if they had not undergone speech therapy. These items were verified in a questionnaire answered by parents and teachers. According to the established criteria, 417 participants were excluded. The final sample consisted of 857 children with typical acquisition, being 399 (46.6%) boys and 458 (53.40%) girls, divided into age groups every 6 months.

### Procedures and assessments

All participants authorized to participate in this study initially underwent a brief conversation with the researcher and were individually assessed using the Phonological Assessment Instrument (INFONO Software)^(19)^. The INFONO software is a standardized instrument for the South of Brazil. Data collection took place in schools during the two school semesters in the same year.

During the INFONO software assessment, each participant was asked to name 84 “animated” images, containing all BP segments in different syllable and word positions. Each image was used to obtain the production of a target word that was obtained by a key question to facilitate the production of the target word, such as: “Does the big bad wolf live in...?” (forest), “What animal is this?” (frog), etc. Other key questions could be asked to obtain the target word and, when necessary, the production of the target was requested by delayed imitation (it was rarely necessary to use imitation, as the target words are easily accessible lexically).

The entire speech sample was recorded in the INFONO software during the assessment. After administering the assessment, it was possible to access the recording and transcripts of the child's production in the software itself. The phonological assessment was carried out in a silent room provided by the school. The assessments were performed by a group of four speech therapists, composed of three doctoral students and a master's student with experience in the field of phonological acquisition and trained in the use of the assessment software. The result of the assessments in the software was performed automatically by the software at the end of the assessment.

The typical phonological acquisition was judged, first, from the conversation with the participant and confirmed after the application of the INFONO software by analyzing the results. Only data from children with typical phonological acquisition remained in the research. 417 participants were excluded for having atypical phonological acquisition, lisp, interposition of the tongue between the teeth, distortion of the segments, among other reasons mentioned in the inclusion criteria.

The reliability of the evaluators' transcripts was examined for 14% of the total sample, that is, the audio recording of 120 children. These recordings were transcribed again independently by a fifth evaluator, called an expert (a speech therapist experienced in the field of phonology) and then a comparison was performed. The reliability of the transcripts between the two evaluators (an original evaluator and the expert) was 95.3%. The transcript from the original evaluator was used to perform the data analysis of this study.

### Data analysis

The software used in the statistical analysis was SPSS (version 23.0). For descriptive statistics, the mean, standard deviation and percentage values were extracted. For each BP segment, the correct production average was calculated in simple and complex onset position and coda position by age group. This result was used for the elaboration of the graph in the phonological acquisition curve format.

For the interpretation of the graphs, the analysis criteria proposed in one study were used^(20)^: a segment was considered acquired when it occurs in 80% or more of the possibilities (in blue in the graph), partially acquired when it occurs in 40-79% of the time (in green on the graph) and not acquired when it occurs 0-39% of the times (in red on the graph). Also, the domain of the segment was considered when the percentage of correct production was equal to or greater than 90% (in gray on the graph).

Then, a comparison of the production of segments in simple and complex onset position and coda position in different age groups was carried out. The correct production average between the age groups was compared using the ANOVA One Way test and an analysis of multiple comparisons using the Post-Hoc Games-Howel test. The results were considered significant when p ≤ 0.05.

## RESULTS

The acquisition curve in simple onset position, of all segments of the stop (/p, b, t, d, k, g/) and nasal (/m, n, ɲ/) classes showed a “ceiling effect” in the percentages, that is, percentages above 95% of correct production already at 3 years old. This means that the domain of these segments was prior to the age analyzed. From 3.0 onwards, the acquisition percentages of these segments remained linear, without showing fluctuations with age, which is why the results will not be presented in graphs. These findings suggest that stops and nasals are the first sounds to be acquired in BP and this development occurs early during the phonological acquisition process.

**Figure 1:**
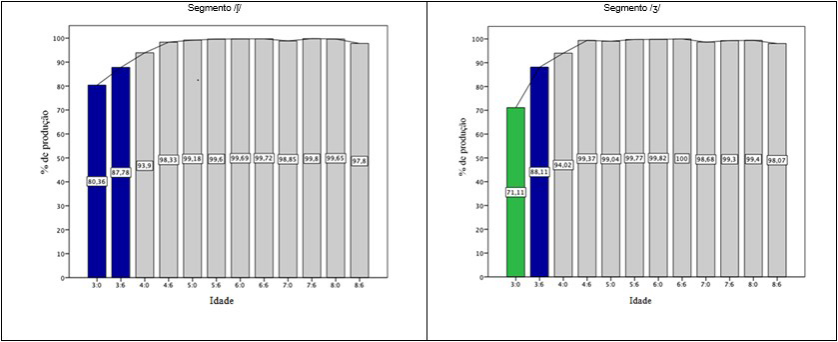
Percentage of correct production of fricative segments in Simple Onset position

The segments of the fricative class (/f, v, s, z/) also had a “ceiling effect” in the results, this effect means that the segments reached a precision rate above 93% of correct production at 3.0 years old (domain age). The segments /ʃ/ and /ʒ/ were the last to be acquired, /ʃ/ at 3.0 and /ʒ/ at 3.6, but the domain of both was at 4.0 years old. In Figure 1, it is possible to note these differences when comparing the graphs of the acquisition curves of these segments.

Caption: Green, partially acquired segment; blue, acquired segment; gray, segment domain. Source: Own elaboration

**Figure 2:**
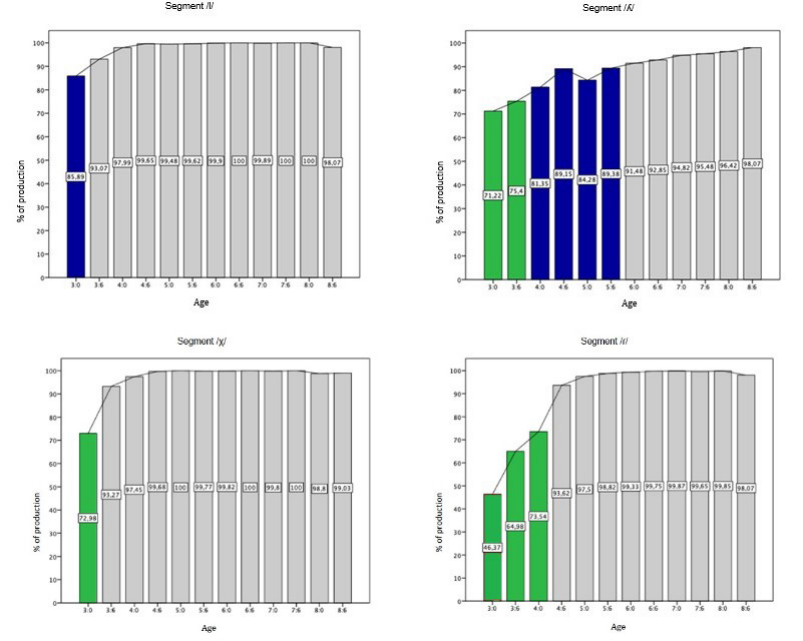
Percentage of correct production of segments (liquids and tap) in Simple Onset position

The segments of the liquid class and the tap /ɾ/ were the last to be acquired in BP. The first segment was /l/, acquired at 3.0 years and the domain at 3.6; followed by the /x/ segment, segment, whose acquisition and domain age were 3.6; The production of /ʎ/ showed the greatest fluctuations, this segment was acquired at 4.0 and the domain only at 6.0 years old. The tap /ɾ/ was acquired at 4.6, the domain also occurred at this age (Figure 2).

Caption: Green, partially acquired segment; blue, acquired segment; gray, segment domain. Source: Own elaboration

**Figure 3:**
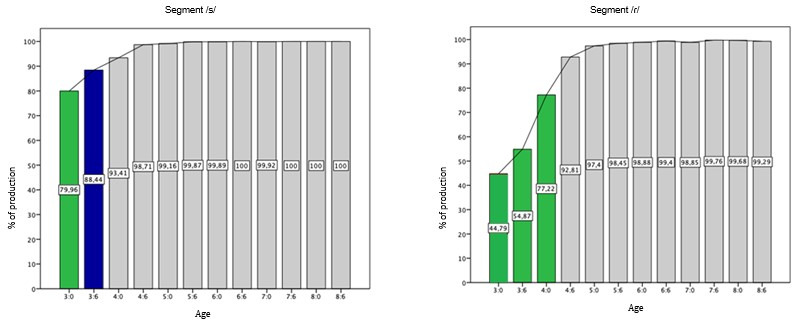
Percentage of correct production of segments in Coda position

The segments that occur in the Coda position in BP were acquired in the following order: /N/ and /L/, followed by /S/, and then /ɾ/. The graphs of /N/ and /L/ showed “ceiling effect”, that is, percentage of correct production above 94%. This means that the acquisition of these segments in the coda structure occurred before the age of 3.0 years, remaining linear, with no fluctuations in the acquisition after that age. The segment /S/ in coda position was acquired at 3.6 and the domain at 4.0, while /ɾ/ was acquired at 4.6, which is also the domain age (Figure 3).

Caption: Green, partially acquired segment; blue, acquired segment; gray, segment domain. Source: Own elaboration

**Figure 4:**
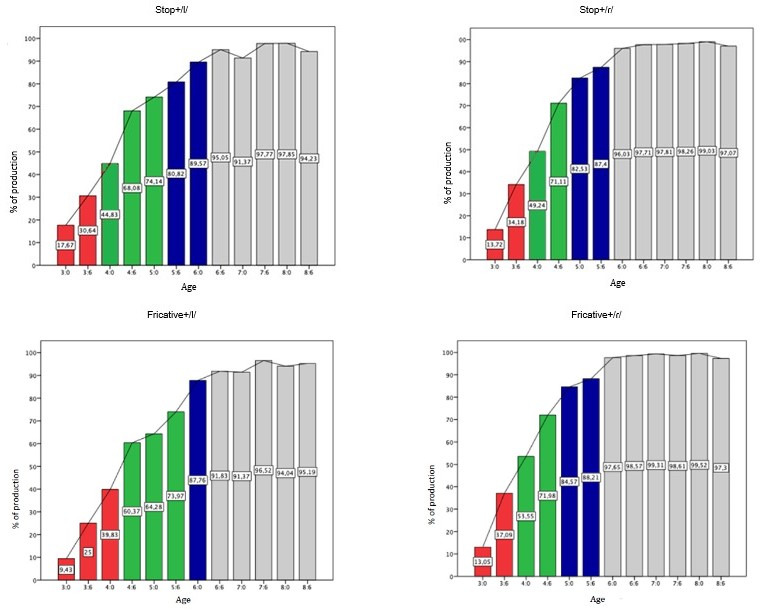
Percentage of correct production of the Onset Complex position

In relation to the complex onset, the production percentages gradually increased over time, with minimal fluctuations (Figure 4). The order of acquisition of the complex onset was Fricative+/ɾ/ and Stop+/ɾ/, followed by Stop+/l/ and, finally, Fricative+/l/. Regarding the acquisition ages, the Fricative+/ɾ/ and Stop+/ɾ/ structures were acquired at 5.0 and the domain at 6.0. Stop+/l/ was acquired at 5.6 and domain occurred at 6:6, while Fricative+/l/ was acquired at 6.0 and domain at 6.6.

Caption: Red, not acquired segment; green, partially acquired segment; blue, acquired segment; gray, segment domain. Source: Own elaboration

Table 1 shows the descriptive analysis (means and standard deviations) and the comparison of performance between the average percentage of correct production of the segments and age for those who did not show a “ceiling effect” in the results. ANOVA showed a significant difference for the production of these segments in simple and complex onset position and coda position in comparison with ages. Also in Table 1, the Post-Hoc analysis showed between which ages there was a statistical difference for all segments that did not show “ceiling effect” in the results, they are /ʃ, ʒ, l, x, ʎ, ɾ/ in onset position, /N, L/ in coda position and complete onset position (Fricative+/ɾ/, Stop+/ɾ/, Fricative+/l/, Stop+/l/. For example, for the segment /ʒ/ the ANOVA showed the existence of a statistically significant difference among the age ranges (F= 24.17; p= .000), specifically. The post hoc analysis performed showed that this difference is between the 3.0 – 3.5 and the 4.0 – 4.5 group. This result confirms that the complete domain of the /ʒ/ segment was achieved in the age group of 4.0 – 4.5 years old.

**Table 1:** Mean and standard deviation when comparing performance among age groups

	Age Group									
Segments	3;0 – 3;5	3;6 – 3;11	4;0 – 4;5	4;6 – 4;11	5;0 – 5;5	5;6 – 5;11	6;0 – 6;5	6;6-6;11	7;0 – 7;5	Anova
	(*n*= 53)	(*n*= 62)	(n= 59)	(n= 53)	(n= 70)	(n= 73)	(*n*= 94)	(*n*= 98)	(*n*= 87)	
	M	M	M	M	M	M	M	M	M	
	(SD)	(SD)	(SD)	(SD)	(SD)	(SD)	(SD)	(SD)	(SD)	
*Onset*										
/ʃ/	80.36 (27.95)^a^	87.78	93.90	98.33						F= 16.37
		(24.10)^a^	(15.76)^a,b^	(4.70)^b^						p= 0.000
/ʒ/	71.11 (35.39)^a^	88.11 (24.63)^a,b^	94.02	99.37						F= 24.17
			(17.18)^b,c^	(4.57)^c^						p= 0.000
/χ/	72.98 (33.64)^a^	93.27	97.45	99.68						F= 28.30 p=0.000
		(18.95)^b^	(8.65)^b^	(2.28)^b^						
/l/	85.89 (21.79)^a^	93.07 (16.27)^a,b^	97.99	99.65						F= 16.57
			(4.48)^b^	(1.74)^b^						p= 0.000
/ʎ/	71.22 (23.20)^a^	75.40 (27.34)^a,b^	81.35 (18.33)^a,b,c^	89.15 (13.43)^c,d^	84.28 (14.22)^b,c^	89.38 (13.12)^c,d^	91.48 (11.90)^d^	92.85 (11.35)^d^	94.82 (10.18)^d^	F= 21.53
										p= 0.000
/ɾ/	46.37 (35.88)^a^	64.98 (37.44)^a,b^	73.54	93.62	97.28	98.82				F= 57.88
			(39.88)^b^	(18.12)^c^	(6.24)^c^	(3.65)^c^				p= 0.000
Coda										F= 30.59
										p= 0.000
/s/	79.96	88.44	93.41	98.71						
	(21.98)^a^	(19.45)^a,b^	(14.66)^b,c^	(8.29)^c^						
/ɾ/	44.79	54.87	77.22	92.81	97.47					F= 79.12
	(33.27)^a^	(36.08)^a^	(32.20)^b^	(20.52)^b,c^	(7.18)^c^					p=0.000
*Onset* Complex										
Stop+/l/			44.83	68.08	74.14	80.82	89.57	95.05		F= 80.00
			(39.29)^a^	(35.26)^a,b^	(31.13)^b,c^	(27.16)^c,d^	(22.47)^d,e^	(14.72)^e^		p= 0.000
Stop+/ɾ/			49.24	71.11	82.53	87.40	96.03			F= 111.27
			(41.01)^a^	(35.25)^a,b^	(28.55)^b^	(22.77)^b^	(10.71)^c^			p= 0.000
Fricative+/l/					64.28	73.97	87.76	91.83		F= 68.48
					(40.05)^a^	(33.44)^a,b^	(23.96)^b,c^	(19.91)^c^		p= 0.000
Fricative+/ɾ/				71.98	84.57	88.21	97.65			F= 88.02
				(39.05)^a^	(29.32)^a^	(24.90)^a,b^	(12.04)^b^			p= 0.000

Caption: *n:* number of participants; M: mean; SD: Standard Deviation. Different letters differ statistically (Teste Post-Hoc Games-Howel). Source: Own elaboration

## DISCUSSION

This study presented the acquisition curves of the BP segments in simple and complex onset position, and coda position according to the classification of the segments in: not acquired, partially acquired, acquired and the domain in the children's speech. The domain of a segment was proposed in this work as the result of a final phonological acquisition process. This evidences that the child generalized the segment in speech, that is, achieved the maximum production.

The correct production of segments evolved with age for all segments without simple and complex onset, and coda, showing that phonological acquisition occurred gradually with advancing age. With regard to age group, the older the age, the greater the maturation of the children's speech system, allowing more complex segments to be produced correctly^(21,22)^. Children with typical acquisition seem to be towards the target production of the segment, that is, seeking production similar to that of adults^(23)^.

In this study, it was verified, in Simple Onset position, that the acquisition and domain of the segments /p, b, t, d, k, g, m, n, ɲ, f, v, s, z/ was before the 3:0 years old. This finding corroborates the results of previous studies^(11,15)^, carried out in Rio de Janeiro and São Paulo, respectively, which identified a similar pattern in the acquisition of nasal and fricative segments (/f, v, s/). Another study^(9)^ carried out in Rio Grande do Sul found that the period of acquisition of stops and nasals (/p, b, t, d, k, g, m, n, ɲ/) was before 2:0 and /z/ at 2 years old. This is possible, since, at 3:0 years old, these segments presented a correct production percentage above 90% (segment domain). However, the results differed in relation to the age of acquisition of the /z/, segment, in which the study^(11)^ proved to be later (3:0-3:11). The difference in this finding can be attributed to regional variations (sample composition), as these studies^(9,11,15)^ were conducted in different regions/states of Brazil. In addition, the methodological criteria adopted were different from those followed in this research, for example, the use of different instruments (test) for phonological assessment.

The acquisition age of the other segments in BP was: /ʃ/ and /l/ at 3:0, /ʒ/ and /x/ at 3:6, /ʎ/ at 4:0, /ɾ/ at 4:6. The domain age was at 4:0 for /ʃ/ and /ʒ/; at 3:6 for /l/ and /x/; at 4:6 for /ɾ/; and at 6:0 for /ʎ/. Regarding the fricatives /ʃ/ and /ʒ/, it was noted that they were the last segments to be acquired within the fricative class, probably because they are more complex. A study^(12)^ from Pernambuco suggested that these segments (/ʃ, ʒ/) were acquired at age 3:6, while another study^(10)^ from Rio de Janeiro suggested that these segments can be acquired later, /f, v, s, z, ʃ/ between 4:0 and 4:11 and /ʒ/ at 5:0. In the state of São Paulo, a study^(15)^ reported the acquisition of these segments (/ʃ, ʒ/) at 3:0 years old.

The segments of the liquid class /x, ʎ, l/ and the tap /ɾ/ were acquired later. This was reported in other studies^(3,9,11,24)^. For the acquisition of these segments, a combination of features is necessary, for example, [+approx., +cont.] for the representation of /ɾ/. This demonstrates the complexity of these segments, requiring greater phonological skills for production, which is generally achieved at older ages. Thus, the hypothesis that phonological acquisition occurs gradually (from least to most complex) was confirmed, with these segments being the most complex and with later acquisition.

Regarding the acquisition ages, in this study, the order identified was firstly /l/ at 3:0 (acquisition age) and the domain at 3:6; followed by /x/ at 3:6 (same age of acquisition and domain), /ʎ/ acquired at 4:0 and domain only occurred at 6:0, and /ɾ/ at 4:6 (same age of acquisition and domain). These results differ a little from what is found in the literature: a study^(10)^ with children in Rio de Janeiro found that /l/, /ʎ/ and /x/ were acquired at 3:0 and /ɾ/ at 4:0; another^(9)^ verified that /l/ was acquired between 2:8 and 3:0, /x/ at 3:4, /ʎ/ at 4:0 and /ɾ/ at 4:2; yet another^(11)^ refer to the acquisition of /l/ and /x/ at 3:0 and of /ʎ/ and /ɾ/ at 5:0. In a study^(15)^ carried out in São Paulo, the acquisition of /ɾ/ and /l/ was at 3:0; /ʎ/, at 3.7 and /x/ at 4:1. Thus, a variability as to the age of correct production of all segments of the liquids and tap /ɾ/ class is noted, however, the segment /ʎ/ showed the most variations. This can be explained by the sociolinguistic and cultural characteristics in the production of one of the INFONO Software's target words. The target word “*colher”* [ku’ʎƐɾ] (spoon) is familiar to young children, but it is often pronounced as [ku’jƐɾ] for sociocultural reasons and linguistic variations. And, as mentioned above, there are different methodological criteria used among the studies, including the phonological assessment tool.

It is noteworthy here that several of the studies^(9,25)^ that present the age of acquisition of segments were carried out with small samples and this may not represent the exact age of phonological acquisition for the majority of the population, requiring care when generalizing the results. For example, in the graph of the segment /ʎ/, it can be noted that at 3:0 years old the average correct production was 71.22%, one child may have acquired this segment at 3:0 years old and another only at 4:0 years old. Therefore, studies with larger samples can provide more reliable acquisition ages, as these samples include greater variability in the production of the segments. This difference was discussed in relation to /ʎ/, but it can be performed for other segments that show variability in terms of age of acquisition among studies.

For the /ɾ/, segment, the same age of acquisition and domain was noted, at 4:6, however, when observing the acquisition curve graph, correct productions were verified from 3:0 years old. Thus, analyzing the acquisition curve is essential, as some children start to produce the segment correctly before others, until, at a certain age, the vast majority need to produce the segment properly.

The acquisition takes place gradually, as seen in the curve, until the child reaches the domain, in which a percentage of correct production equal to or greater than 90% of correct production is noted, with no or minimal change/error in production. For example, a 5:0-year-old child who, in the phonological assessment, found that he/she had 65% of correct production for the segment /ɾ/, when looking at the graph, it was noted that this percentage is close to what is expected at 3.6 years old (already indicative of delay or alert in the progress of the acquisition) and that at 5, this segment should have a percentage of correct production greater than 90%, indicating domain of the phoneme. Thus, the acquisition curve makes it possible to verify in a more dynamic way whether a child is within the expected range for his/her age or to infer delays or alerts for delays in phonological acquisition.

Regarding coda, the segments /N/ and /L/ were acquired and dominated before 3:0, /S/ at 3:6 and /ɾ/ at 4:6. These findings suggest an acquisition a little later than that reported by a study^(9)^ that identified a similar order of acquisition, but at an earlier age for the segments /S/ and /ɾ/ (/S/ between 2:6 and 3:0 and /ɾ/ at 3:10).

Thus, this study confirmed that the BP segments are acquired in the following order: stops and nasals, followed by fricatives and, finally, liquids/tap. These findings corroborate previous studies on the acquisition of BP segments^(3,10,24)^.

The Complex Onset analysis showed that acquisition and domain occur in different ways among the different possibilities (Fricative+/ɾ/, Fricative+/l/, Stop+/ɾ/, Stop+/l/) of realization. The structures composed by Fricative+/ɾ/ and Stop+/ɾ/ were acquired first, at, at 5:0, and the domain was at 6:0. In turn, the structures composed of Stop or Fricative+/l/ were acquired later, the Stop+/l/ was acquired at 5:6 and the domain at 6:6 and the Fricative+/l/ was acquired at 6:0 and the domain at 6:6. One study^(10)^ suggests that the complex onset structure can be acquired between 4:0 and 4:6. Other studies^(15,26)^ report that the groups consisting of /ɾ/ are acquired before those formed by /l/, which corroborates the findings of this study, as well as the domain of structure, with similar ages. Slightly divergent, the results of the study^(10)^ which verified that the structure composed by /l/ was acquired at 4:0 and the structure with /ɾ/ at 5:0, showing that the structure composed by /l/ was acquired before.

Due to the divergences mentioned throughout the study, it is necessary and important to carry out more studies on the phonological acquisition of BP. Thus, it is expected that this work with analysis of phonological acquisition of typical children contributes in a theoretical and practical way, allowing the identification of possible changes during this chronology and even in the identification of children with atypical phonological acquisition^(1)^. For the speech therapist, this is essential, as it allows the assessment, intervention and treatment of children with atypical phonological acquisition before they reach the period for literacy. Atypical children seem to be more immature with regard to motor acquisition, especially in the production of speech sounds when compared to typical children^(27)^. Still, it is suggested that future studies on phonological acquisition be carried out with larger samples so that results can be generalized to the Brazilian population with its different characteristics.

The curve format, to represent phonological acquisition, is a tool that helps in the dynamics to understand the phonological acquisition process, as the child starts from 0% to 90% correct production or more when he/she reaches a certain age. In the curve format, the easy visualization of the percentages of correct production in different age groups, helps in comparing the children themselves, with similar age groups, measuring what would be expected for their age.

It is important to know parameters such as age of acquisition and domain of phonemes to establish whether or not there is a delay in acquisition, but it is also equally important to analyze this progression. For example, a 4:6-year-old child who, in the phonological assessment, was found to have 65% of correct production for the segment /ɾ/. If only the parameters are noted, it can be concluded that the child is at the age of acquisition of this segment, and that he/she has already started to perform it correctly, but there is a risk of concluding that there is no delay in the acquisition of this segment. However, when looking at the acquisition curve, it is noted that the production percentage of 65% is close to what is expected at 3:6 years old (which could be a warning sign for a delay in the acquisition of this segment in relation to its peers). Thus, analyzing the acquisition curve allows the clinician to confirm whether a child is within the expected range for his/her age or to infer delays in phonological acquisition.

Studies^(5,28)^ report that, in clinical practice, normative acquisition data can help speech therapists to differentiate children with typical development or those with developmental delay. It is believed that the speech therapist should consider, in addition to the acquisition and domain parameters, the percentage of correct production of the curve to assist in diagnosis and therapy, proposing speech therapy actions that help in the acquisition of this segment.

The findings of this study must be interpreted considering some limitations, such as: demographic limitations of the sample, which was recruited only in the southern region of Brazil and children under 3:0 were not evaluated, as some segments have acquisition and domain ages previous.

## CONCLUSION

In this article, the phonological acquisition of BP segments in simple and complex onset position and coda position was analyzed using the acquisition curve. The results show that, in simple onset position, at 3:0, the stops, nasals and fricatives /f/, /v/, /s/ and /z/ were acquired and dominated. The acquisition of /ʃ/ and /l/ was at 3:0, /ʒ/ and /x/ at 3:6, /ʎ/ at 4:0, /ɾ/ at 4:6. However, the domain of these segments was: /ʃ/ and /ʒ/ at 4:0, /l/ and /x/ at 3:6, /ɾ/ at 4:6 and /ʎ/ at 6:0. In coda position, the acquisition and domain of /N/ and /L/ was before 3:0, /S/ was acquired at 3:6 and the domain was at 4:0 and /ɾ/ at 4:6 (same age of acquisition and domain). In complex onset position, the structures composed of Fricative+/ɾ/ and Stop+/ɾ/ were acquired at 5:0 and the domain at 6:0, the Stop+/l/ was acquired at 5:6 and the domain at 6:6 and Fricative+/l/ was acquired at 6:0 and domain at 6:6.

It is noteworthy that acquisition and domain for some children may occur before these ages found, and it is important to analyze the acquisition curve. This has significant implications for the assessment and diagnosis of children with suspected speech sound disorders.

In addition, it is noted that, as children become older, the average correct production of the segments increases, enabling the increment of new segments in their phonological inventory.
