# Supplementary material for: Segmental acquisition of Brazilian Portuguese: onset simple, complex and coda
Source: Codas. 2022 Jan 12;34(3):e20200439. doi: 10.1590/2317-1782/20212020439 (PMC9851189; doi:10.1590/2317-1782/20212020439)
Supplement: Translation

# Translation: Aquisição segmental do Português Brasileiro: *onset* simples, complexo e coda

**Authors:** Marizete Ilha Ceron, Simone Nicolini de Simoni, Gabriel Agustín Urrutia Urrutia, Márcia Keske-Soares

**Affiliations:** 1 Prefeitura de Santa Cruz do Sul - Santa Cruz do Sul (RS), Brasil.; 2 Programa de Pós-graduação em Distúrbios da Comunicação Humana, Universidade Federal de Santa Maria – UFSM - Santa Maria (RS), Brasil.; 3 Programa de Pós-graduação em Distúrbios da Comunicação Humana, Universidade Federal de Santa Maria – UFSM - Santa Maria (RS), Brasil.; 4 Universidad de Talca - Talca, Region del Maule, Chile.; 5 Laboratório de Fala – LabFala, Departamento de Fonoaudiologia, Universidade Federal de Santa Maria – UFSM - Santa Maria (RS), Brasil.

**Keywords:** Fala, Aquisição Fonológica, Linguagem Infantil, Criança, Fonologia

## Abstract

**Objetivo:**

Apresentar e analisar a curva de aquisição segmental do Português Brasileiro em *onset* simples, complexo e coda.

**Método:**

Participaram 857 crianças com aquisição fonológica típica, com idades entre 3:0 e 8:11, divididos em faixas etárias de 6 em 6 meses. Os participantes foram avaliados, por meio do *software* INFONO de avaliação fonológica. Após a análise dos resultados foram elaboradas curvas de aquisição dos segmentos analisados nas diferentes estruturas (*onset* simples, complexo e coda).

**Resultados:**

Observou-se que, em *onset* simples, alguns segmentos estavam adquiridos antes dos 3:0 (plosivas, nasais e fricativas /f, v, s, z/). Os segmentos /ʃ/ e /l/ foram adquiridos aos 3:0, /ʒ/ e /x/ aos 3:6, /ʎ/ aos 4:0, /ɾ/ aos 4:6; em coda, /N/ e /L/ foram adquiridos antes dos 3:0, /S/ aos 3:6 e /ɾ/ aos 4:6; em *onset* complexo, as estruturas compostas por Fricativa+/ɾ/ e Plosiva+/ɾ/ foram adquiridas aos 5:0, Plosiva+/l/ aos 5:6 e Fricativa + /l/ aos 6:0.

**Conclusão:**

Analisar a curva de aquisição é fundamental, pois fornece referência sobre a idade de aquisição dos segmentos nas diferentes estruturas silábicas. A curva de aquisição contribui para a identificação precoce de atrasos no processo de aquisição fonológica possibilitando um encaminhamento oportuno para a intervenção fonoaudiológica.

## INTRODUÇÃO

A aquisição fonológica do Português Brasileiro (PB) é um tema relevante a ser pesquisado e discutido em meio científico. Este conhecimento proporciona o entendimento sobre o processo de aquisição dos segmentos da língua e como acontece para diferentes crianças^(1)^.

O inventário de consoantes do PB é constituído por 19 segmentos, do ponto de vista fonológico é distribuído em classe de sons, formando as plosivas (/p, b, t, d, k, g/), nasais (/m, n, ɲ /), fricativas (/f, v, s, z, ʃ, ʒ/), líquidas (/x, ʎ, l/) e o *tap* /ɾ/. Esses segmentos são ainda divididos conforme a estrutura silábica da língua em *onset* simples, complexo e coda^(2)^.

A aquisição fonológica das consoantes do PB, inicia-se de forma gradual e progressiva. Estima-se que aproximadamente aos 5 anos de idade a criança apresente o inventário fonológico completo ou próximo ao do adulto. A ordem de aquisição e desenvolvimento das classes de sons é plosivas e nasais, seguido de fricativas e líquidas incluindo o *tap* /ɾ/, essa última classe é considerada de domínio mais tardio^(3,4)^. Essa mesma ordem de aquisição também é referida em estudos internacionais^(5,6)^.

O *onset* complexo é a estrutura de aquisição mais tardia, por apresentar maior complexidade de produção^(7)^. Um estudo^(8)^ mostrou não existir diferença na idade de aquisição entre as estruturas compostas por obstruinte+/ɾ/ e obstruinte+/l/ e que o *onset* complexo que foi adquirido entre 4:0 e 4:6.

É importante conhecer e entender o processo de aquisição fonológica típica para conseguir identificar, diagnosticar e tratar crianças com suspeitas de alterações nessa cronologia de aquisição. Na literatura^(9-15)^ observam-se diferenças nas idades relacionadas à aquisição dos segmentos tanto na estrutura de *onset* simples quanto na de *onset* complexo. Ressalta-se maior discrepância nos achados relacionados ao *onset* complexo. Essas diferenças em relação à idade de aquisição podem acontecer devido a:

- Variações metodológicas das pesquisas, por exemplo, a utilização de diferentes faixas etárias, algumas utilizam faixas de 12 meses^(10,11)^ outras de 6 meses^(3,8)^, outro de 4 meses^(14)^, outra é longitudinal^(2,13)^. A aquisição fonológica é gradual e rápida nos primeiros anos de vida, então analisar faixas muito grandes pode não apresentar a real idade de aquisição e domínio de um determinado fonema;
- Formas de coletas dos dados, por exemplo, alguns estudos^(10,11)^ elaboraram listas específicas que foram eliciadas mediante nomeação de figuras isoladas, outro^(8)^ com a nomeação de figuras de instrumento fonológico não padronizado, ainda outros^(3,14)^ pela nomeação de figuras a partir de instrumento fonológico padronizado (instrumento que foi elaborado seguindo critérios psicométricos para serem utilizados) e outro^(2)^ pela produção espontânea com auxílio de objetos. Cada vez mais sabe-se a importância de utilizar instrumentos padronizados no processo de avaliação e que esses instrumentos apresentem critérios psicométricos de validade e fidedignidade para a confiabilidade dos resultados da avaliação^(16,17)^. Assim, a forma de coleta dos dados é um fator importante para que estudos possam ser comparados;
- Amostras reduzidas. O tamanho da amostra em cada faixa etária é importante para que a informação possa ser generalizada para uma população. Alguns estudos nacionais foram realizados com um número extremamente pequeno de crianças^(2,8,13)^ enquanto outros com amostras um pouco mais expressivas^(10,11)^. Acredita-se que a avaliação fonológica realizada interfira diretamente na amostra por ser uma avaliação cuja análise é demorada, demandando de tempo, o que dificulta realizar em grande quantidade. Porém, o tamanho da amostra é um fator destacado na literatura^(17,18)^ no sentido de que os grupos de participantes devem ser grandes o suficiente para garantir comparações de subgrupos com as pontuações da população em geral. Estudos internacionais que analisam a aquisição mencionam amostras superiores^(5,6)^;
- Pesquisas realizadas em diferentes regiões do Brasil (heterogeneidade da língua). A língua é a mesma, mas com traços peculiares de cada região, podendo variar um pouco a idade de aquisição e domínio de alguns fonemas.

Dessa forma, acredita-se que essas diferenças podem trazer divergências importantes nas idades de aquisição e domínio dos segmentos do PB. Isso torna imprescindível a realização de mais estudos sobre a aquisição fonológica do PB com faixas etárias menores, com instrumentos padronizados/normatizado para a população, com um maior número de sujeitos por faixa etária e para manter os dados atualizados, pois a língua é dinâmica e está em constante mudança.

Espera-se que a curva de aquisição dos segmentos realizada a partir de dados de aquisição fonológica de crianças típicas possa determinar se o desenvolvimento da fala de uma criança progride normalmente ou está atrasado nesse processo de aquisição e, assim, auxiliar no diagnóstico e intervenção antes do período de alfabetização.

Diante do exposto, o objetivo deste trabalho foi apresentar e analisar a curva de aquisição segmental do PB em *onset* simples, complexo e coda. Para tanto, tem-se as seguintes hipóteses: a aquisição fonológica ocorre de maneira dinâmica e gradual, iniciando pelos segmentos menos complexos até atingir os mais complexos; um segmento começa ser produzido (idade de produção), segue até a aquisição (idade de aquisição) e por fim, atinge o domínio completo na fala (idade de domínio); que a idade de produção, aquisição e domínio dos segmentos sejam diferentes.

## MÉTODO

Este estudo é descritivo, transversal e quantitativo. O projeto foi aprovado pelo Comitê de Ética em Pesquisa sob o número 23081.005433/2011-65. Todas as crianças foram autorizadas pelos pais e/ou responsáveis para participarem da pesquisa por meio da assinatura do Termo de Consentimento Livre e Esclarecido. Foram respeitadas as Diretrizes de Pesquisa e Saúde, conforme a Resolução 466/2012.

### Participantes

A amostra foi de conveniência. Foram selecionadas 12 escolas de duas cidades do Rio Grande do Sul (Brasil) (8 públicas e 4 privadas), um total de 1806 crianças foram convidadas a participar do estudo, das quais 1274 (70%) foram autorizadas pelos pais a participar. Todas as crianças tinham idades entre 3 anos e 8 anos e 11 meses.

Os critérios de elegibilidade foram: crianças monolíngues do PB que apresentam aquisição fonológico típico, com ausência de histórico ou suspeita de alterações auditivas e neurológica se que não tivessem realizado terapia fonoaudiológica. Esses itens foram verificados em um questionário respondido pelos pais e professores. Conforme os critérios estabelecidos, 417 participantes foram excluídos. A amostra final foi constituída por 857 crianças com aquisição típica, sendo 399 (46,6%) meninos e 458 (53,40%) meninas, divididas em faixas de idade de 6 em 6 meses.

### Procedimentos e avaliações

Todos os participantes autorizados a participar deste estudo passaram, inicialmente, por uma breve conversa com o pesquisador e foram avaliados individualmente por meio do Instrumento de Avaliação Fonológica (*Software* INFONO)^(19)^. O *software* INFONO é um instrumento padronizado e normatizado para o Sul do Brasil. A coleta de dados ocorreu nas escolas durante os dois semestres escolares no mesmo ano.

Durante a avaliação pelo *software* INFONO, cada participante foi convidado a nomear 84 imagens “animadas”, contendo todos os segmentos do PB em diferentes posições de sílaba e palavra. Cada imagem foi usada para obter a produção de uma palavra-alvo que foi obtida por uma pergunta-chave para facilitar a produção da palavra-alvo, tais como: “O lobo mau mora na...?” (floresta), “Que animal é este?” (sapo), etc. Outras perguntas-chave poderiam ser realizadas para obter a palavra-alvo e, quando necessário, a produção do alvo foi solicitada por imitação retardada (raramente foi necessário utilizar a imitação, pois as palavras-alvo são de fácil acesso lexical).

Toda a amostra de fala foi registrada no *software* INFONO durante a avaliação. Após a administração da avaliação foi possível acessar a gravação e as transcrições da produção da criança no próprio *software*. A avaliação fonológica foi realizada em uma sala silenciosa cedida pela escola. As avaliações foram realizadas por um grupo de quatro fonoaudiólogas, composto de três doutorandas e uma mestranda com experiência na área de aquisição fonológica e treinadas para a utilização do *software* de avaliação. O resultado das avaliações no *software* foi realizado de forma automática pelo *software* no final da avaliação.

A aquisição fonológica típica foi julgada, primeiramente, a partir da conversa com o participante e confirmada após a aplicação do *software* INFONO pela análise dos resultados. Somente dados de crianças com aquisição fonológica típica permaneceram na pesquisa. Foram excluídos 417 participantes por terem aquisição fonológica atípica, ceceio, interposição da língua entre os dentes, distorção dos segmentos, entre outros motivos referido nos critérios de inclusão.

A confiabilidade das transcrições dos avaliadores foi examinada para 14% da amostra total, ou seja, a gravação de áudio de 120 crianças. Essas gravações foram transcritas novamente de forma independente por um quinto avaliador, denominado perito (fonoaudiólogo experiente na área de fonologia) e então foi realizada a comparação. A confiabilidade das transcrições entre os dois avaliadores (um avaliador original e o perito) foi de 95,3%. A transcrição do avaliador original foi utilizada para a realização das análises de dados deste estudo.

### Análise de dados

O *software* utilizado na análise estatística foi o SPSS (versão 23.0). Para a estatística descritiva, extraíram-se os valores de média, desvio padrão e percentuais. Para cada segmento do PB, foi calculada a média de produção correta em *onset* simples, complexo e coda por faixa etária. Este resultado foi utilizado para a elaboração do gráfico no formato de curva de aquisição fonológica.

Para a interpretação dos gráficos, utilizaram-se os critérios de análise propostos em um estudo^(20)^: um segmento foi considerado adquirido quando ocorre em 80% ou mais das possibilidades (em azul no gráfico), parcialmente adquirido quando ocorre de 40-79% das vezes (em verde no gráfico) e não adquirido quando ocorre de 0-39% das chances (em vermelho no gráfico). Ainda o domínio do segmento foi considerado quando o percentual de produção correta foi igual ou superior a 90% (em cinza no gráfico).

Em seguida, realizou-se uma comparação da produção dos segmentos em *onset* simples, complexo e coda nas diferentes faixas etárias. Contrastou-se a média de produção correta entre as faixas de idade por meio da prova ANOVA One Way e uma análise de comparações múltiplas pelo teste Post-Hoc Games-Howel. Os resultados foram considerados significantes quando p ≤ 0,05.

## RESULTADOS

A curva de aquisição em *onset* simples, de todos os segmentos das classes das plosivas (/p, b, t, d, k, g/) e nasais (/m, n, ɲ/) apresentaram “efeito teto” nos percentuais, ou seja, percentuais acima de 95% de produção correta já aos 3;0 anos de idade. Isso significa que o domínio desses segmentos foi anterior à idade analisada. A partir dos 3;0, os percentuais de aquisição desses segmentos mantiveram-se de forma linear sem apresentar oscilações com a idade, motivo pelo qual não serão apresentados os resultados em gráficos. Esses achados sugerem que as plosivas e nasais são os primeiros sons a serem adquiridos no PB e esse desenvolvimento ocorre precocemente durante o processo de aquisição fonológica.

Os segmentos da classe das fricativas (/f, v, s, z/) também apresentaram “efeito teto” nos resultados, esse efeito, significa que os segmentos atingiram uma taxa de precisão acima de 93% de produção correta aos 3;0 anos de idade (idade de domínio). Os segmentos /ʃ/ e /ʒ/ foram os últimos a serem adquiridos, /ʃ/ aos 3;0 e /ʒ/ aos 3;6, mas o domínio de ambos foi aos 4;0 anos de idade. Na Figura 1, é possível observar essas diferenças ao comparar os gráficos das curvas de aquisição desses segmentos.

**Figura 1:** Percentual de produção correta dos segmentos fricativos em *Onset* Simples

Legenda: Verde, segmento parcialmente adquirido; azul, segmento adquirido; cinza, domínio do segmento. Fonte: Elaboração própria

Os segmentos da classe das líquidas e o *tap* /ɾ/ foram os últimos a serem adquiridos no PB. O primeiro segmento foi o /l/, adquirido aos 3;0 anos e o domínio aos 3;6; seguido do segmento /x/, cuja idade de aquisição e de domínio foi 3;6. A produção de /ʎ/ foi a que apresentou maiores oscilações, esse segmento foi adquirido aos 4;0 e o domínio somente aos 6;0 de idade. O *tap* /ɾ/ foi adquirido aos 4;6, o domínio também ocorreu nesta mesma idade (Figura 2).

**Figura 2:** Percentual de produção correta dos segmentos (líquidas e *tap*) em *Onset* Simples

Legenda: Verde, segmento parcialmente adquirido; azul, segmento adquirido; cinza, domínio do segmento. Fonte: Elaboração própria

Os segmentos que ocorrem na posição de Coda no PB foram adquiridos na seguinte ordem: /N/ e /L/, seguidos por /S/, e depois /ɾ/. Os gráficos de /N/ e /L/ mostraram “efeito teto”, ou seja, percentual de produção correta acima de 94%. Isso significa que a aquisição desses segmentos na estrutura da coda ocorreu antes dos 3;0 anos, mantendo-se linear, sem apresentar oscilações na aquisição após essa idade. O segmento /S/ em coda foi adquirido aos 3;6 e o domínio aos 4;0, enquanto /ɾ/ foi adquirido aos 4;6, sendo esta também a idade de domínio (Figura 3).

**Figura 3:** Percentual de produção correta dos segmentos em Coda

Legenda: Verde, segmento parcialmente adquirido; azul, segmento adquirido; cinza, domínio do segmento. Fonte: Elaboração própria

Em relação ao *onset* complexo os percentuais de produção aumentaram gradualmente ao longo do tempo, com mínimas oscilações (Figura 4). A ordem de aquisição do *onset* complexo foi Fricativa+/ɾ/ e Plosiva+/ɾ/, seguido pela Plosiva+/l/ e, por último, Fricativa+/l/. Em relação às idades de aquisição, as estruturas Fricativa+/ɾ/ e Plosiva+/ɾ/ foram adquiridas aos 5;0 e o domínio aos 6;0. A Plosiva+/l/ foi adquirida aos 5;6 e o domínio ocorreu aos 6:6, enquanto a Fricativa+/l/ foi adquirida aos 6;0 e o domínio aos 6;6.

**Figura 4:** Percentual de produção correta do *Onset* Complexo

Legenda: Vermelho, segmento não adquirido; verde, segmento parcialmente adquirido; azul, segmento adquirido; cinza, domínio do segmento. Fonte: Elaboração própria

A Tabela 1 mostra a análise descritiva (médias e desvios-padrão) e a comparação do desempenho entre a média do percentual de produção correta dos segmentos e a idade para os que não apresentaram “efeito teto” nos resultados. A ANOVA mostrou diferença significativa para a produção desses segmentos em *onset* simples, complexo e coda na comparação com as idades. Ainda na Tabela 1, a análise Post-Hoc apresentou entre quais idades houve diferença estatística para todos os segmentos que não apresentaram “efeito teto” nos resultados, são eles /ʃ, ʒ, l, x, ʎ, ɾ/ em *onset*, /N, L/ em coda e *onset* completo (Fricativa+/ɾ/, Plosiva+/ɾ/, Fricativa+/l/, Plosiva+/l/. Por exemplo, para o segmento /ʒ/ a ANOVA mostrou a existência de diferença estatística significativa entre as faixas de idade (F= 24.17; p= .000), especificamente. A análise post hoc realizada mostrou que essa diferença está entre o grupo de 3;0 – 3;5 e o de 4;0 – 4;5. Esse resultado confirma que o domínio completo do segmento /ʒ/ foi alcançado na faixa etária de 4;0 – 4;5 anos.

**Tabela 1:** Média e desvio padrão na comparação do desempenho entre as faixas etária

	Faixa etária									
Segmentos	3;0 – 3;5	3;6 – 3;11	4;0 – 4;5	4;6 – 4;11	5;0 – 5;5	5;6 – 5;11	6;0 – 6;5	6;6-6;11	7;0 – 7;5	Anova
	(*n*= 53)	(*n*= 62)	(n= 59)	(n= 53)	(n= 70)	(n= 73)	(*n*= 94)	(*n*= 98)	(*n*= 87)	
	M	M	M	M	M	M	M	M	M	
	(SD)	(SD)	(SD)	(SD)	(SD)	(SD)	(SD)	(SD)	(SD)	
*Onset*										
/ʃ/	80,36 (27,95)^a^	87,78	93,9	98,33						F= 16,37
		(24,10)^a^	(15,76)^a,b^	(4,70)^b^						p= 0,000
/ʒ/	71,11 (35,39)^a^	88,11 (24,63)^a,b^	94,02	99,37						F= 24,17
			(17,18)^b,c^	(4,57)^c^						p= 0,000
/x/	72,98 (33,64)^a^	93,27	97,45	99,68						F= 28,30 p=0,000
		(18,95)^b^	(8,65)^b^	(2,28)^b^						
/l/	85,89 (21,79)^a^	93,07 (16,27)^a,b^	97,99	99,65						F= 16,57
			(4,48)^b^	(1,74)^b^						p= 0,000
/ʎ/	71,22 (23,20)^a^	75,40 (27,34)^a,b^	81,35 (18,33)^a,b,c^	89,15 (13,43)^c,d^	84,28 (14,22)^b,c^	89,38 (13,12)^c,d^	91,48 (11,90)^d^	92,85 (11,35)^d^	94,82 (10,18)^d^	F= 21,53
										p= 0,000
/ɾ/	46,37 (35,88)^a^	64,98 (37,44)^a,b^	73,54	93,62	97,28	98,82				F= 57,88
			(39,88)^b^	(18,12)^c^	(6,24)^c^	(3,65)^c^				p= 0,000
Coda										F= 30,59
										p= 0,000
/s/	79,96	88,44	93,41	98,71						
	(21,98)^a^	(19,45)^a,b^	(14,66)^b,c^	(8,29)^c^						
/ɾ/	44,79	54,87	77,22	92,81	97,47					F= 79,12
	(33,27)^a^	(36,08)^a^	(32,20)^b^	(20,52)^b,c^	(7,18)^c^					p=0,000
*Onset* Complexo										
Plosiva+/l/			44,83	68,08	74,14	80,82	89,57	95,05		F= 80,00
			(39,29)^a^	(35,26)^a,b^	(31,13)^b,c^	(27,16)^c,d^	(22,47)^d,e^	(14,72)^e^		p= 0,000
Plosiva+/ɾ/			49,24	71,11	82,53	87,4	96,03			F= 111,27
			(41,01)^a^	(35,25)^a,b^	(28,55)^b^	(22,77)^b^	(10,71)^c^			p= 0,000
Fricativa+/l/					64,28	73,97	87,76	91,83		F= 68,48
					(40,05)^a^	(33,44)^a,b^	(23,96)^b,c^	(19,91)^c^		p= 0,000
Fricativa+/ɾ/				71,98	84,57	88,21	97,65			F= 88,02
				(39,05)^a^	(29,32)^a^	(24,90)^a,b^	(12,04)^b^			p= 0,000

Legenda: *n:* número de participantes; M: média; SD: Desvio Padrão. Letras diferentes diferem estatisticamente (Teste Post-Hoc Games-Howel). Fonte: Elaboração própria

## DISCUSSÃO

Este estudo apresentou as curvas de aquisição dos segmentos do PB em *onset* simples, complexo, e coda de acordo com a classificação dos segmentos em: não adquirido, parcialmente adquirido, adquirido e o domínio na fala das crianças. O domínio de um segmento foi proposto neste trabalho como o resultado de um processo final de aquisição fonológica. Isso evidência que a criança generalizou o segmento na fala, ou seja, conquistou o máximo de produção.

A produção correta dos segmentos evoluiu com a idade para todos os segmentos sem *onset* simples, complexo, e coda, mostrando que a aquisição fonológica ocorreu gradualmente com o avanço da idade. No que se refere à faixa etária, quanto maior a idade, maior o amadurecimento do sistema fonoarticulatório das crianças, permitindo que segmentos mais complexos sejam produzidos corretamente^(21,22)^. As crianças com aquisição típica parecem estar em direção à produção-alvo do segmento, ou seja, buscando a produção semelhante à do adulto^(23)^.

No presente estudo, verificou-se, em *Onset* Simples, que a aquisição e domínio dos segmentos /p, b, t, d, k, g, m, n, ɲ, f, v, s, z/ foi antes dos 3:0 anos de idade. Esse achado corrobora os resultados de estudos prévio^(11,15)^, realizado no Rio de Janeiro e São Paulo, respectivamente, que identificou um padrão semelhante na aquisição de segmentos nasais e fricativos (/f, v, s/). Outro estudo^(9)^ realizado no Rio Grande do Sul constatou que o período de aquisição de plosivas e nasais (/p, b, t, d, k, g, m, n, ɲ/) foi antes dos 2:0 e /z/ aos 2 anos. Isso é possível, visto que, aos 3:0 anos, esses segmentos apresentaram um percentual de produção correta acima de 90% (domínio do segmento). No entanto, os resultados diferiram em relação à idade de aquisição do segmento /z/, em que o estudo^(11)^ mostrou ser mais tardia (3:0-3:11). A diferença desse achado pode ser atribuída às variações regionais (composição amostral), pois esses estudos^(9,11,15)^ foram conduzidos em diferentes regiões/estados do Brasil. Além disso, os critérios metodológicos adotados foram diferentes dos seguidos nesta pesquisa, por exemplo, uso de instrumentos (teste) diferentes para avaliação fonológica.

A idade de aquisição dos outros segmentos no PB foi: /ʃ/ e /l/ aos 3:0, /ʒ/ e /x/ aos 3:6, /ʎ/ aos 4:0, /ɾ/ aos 4:6. A idade de domínio foi aos 4:0 para /ʃ/ e /ʒ/; aos 3:6 para /l/ e /x/; aos 4:6 para /ɾ/; e aos 6:0 para /ʎ/. Em relação aos fricativos /ʃ/ e /ʒ/, observou-se que foram os últimos segmentos a serem adquiridos dentro da classe das fricativas, provavelmente por serem mais complexos. Um estudo^(12)^ de Pernambuco sugeriu que estes segmentos (/ʃ, ʒ/) foram adquiridos na idade 3:6, enquanto outro estudo^(10)^ do Rio de Janeiro sugeriu que esses segmentos podem ser adquiridos mais tarde, /f, v, s, z, ʃ/ entre 4:0 e 4:11 e /ʒ/ aos 5:0. No estado de São Paulo, um estudo^(15)^ referiu a aquisição desses segmentos (/ʃ, ʒ/) aos 3:0 anos.

Os segmentos da classe das líquidas /x, ʎ, l/ e o *tap* /ɾ/ foram os de aquisição mais tardia. Isso foi referido em outros estudos^(3,9,11,24)^. Para a aquisição desses segmentos é necessária uma combinação de traços, por exemplo, [+aprox., +cont.] para a representação de /ɾ/. Isso demonstra a complexidade desses segmentos, necessitando maior habilidade fonológica para a produção, o que é geralmente alcançado em idades mais avançadas. Dessa forma, a hipótese de que a aquisição fonológica ocorre de maneira gradual (do menos para o mais complexo) foi confirmada, sendo esses segmentos os mais complexos e de aquisição mais tardia.

Em relação às idades de aquisição, neste estudo a ordem identificada foi primeiramente o /l/ aos 3:0 (idade de aquisição) e o domínio aos 3:6; seguido por /x/ aos 3:6 (mesma idade de aquisição e de domínio), /ʎ/ adquirido aos 4:0 e o domínio ocorreu somente aos 6:0, e /ɾ/ aos 4:6 (mesma idade de aquisição e domínio). Esses resultados diferem um pouco do que é encontrado na literatura: um estudo^(10)^ com crianças no Rio de Janeiro, verificaram que /l/, /ʎ/ e /x/ foram adquiridas aos 3:0 e /ɾ/ aos 4:0; outro^(9)^ verificou que /l/ foi adquirido entre 2:8 e 3:0, /x/ aos 3:4, /ʎ/ aos 4:0 e /ɾ/ aos 4:2; ainda outro^(11)^ referem a aquisição de /l/ e /x/ aos 3:0 e de /ʎ/ e /ɾ/ aos 5:0. Em um estudo^(15)^ realizado em São Paulo, a aquisição do /ɾ/ e /l/ foi aos 3:0; /ʎ/, aos 3;7 e /x/ aos 4:1. Dessa forma, observa-se uma variabilidade quanto à idade de produção correta de todos os segmentos da classe das líquidas e tap /ɾ/, porém, o segmento /ʎ/ foi o que mais apresentou variações. Isso pode ser explicado devido as características sociolinguísticas e culturais na produção de uma das palavras-alvo do *Software* INFONO. A palavra-alvo *colher* [ku’ʎƐɾ] é familiar para crianças pequenas, porém é pronunciado muitas vezes como [ku’jƐɾ] por questões socioculturais e variações linguísticas. E, ainda, conforme mencionando anteriormente, há diferentes critérios metodológicos utilizados entre os estudos, incluindo a ferramenta de avaliação fonológica.

Convém ressaltar aqui que vários dos estudos^(9,25)^ que apresentam a idade de aquisição de segmentos foram realizados com amostras pequenas e isso pode não representar a exata idade de aquisição fonológica para a maioria da população, necessitando de cuidados ao generalizar os resultados. Por exemplo, no gráfico do segmento /ʎ/, pode-se observar que aos 3:0 anos a média de produção correta foi de 71,22%, podendo uma criança ter adquirido esse segmento aos 3:0 anos e outra somente aos 4:0 anos. Por isso, estudos com amostras maiores podem fornecer idades de aquisição mais confiáveis, pois essas amostras contemplam maior variabilidade na produção dos segmentos. Essa diferença foi discutida em relação ao /ʎ/, mas pode ser realizada para outros segmentos que apresentam variabilidade quanto à idade de aquisição entre os estudos.

Para o segmento /ɾ/, observou-se a mesma idade de aquisição e domínio, aos 4:6, porém, ao observar o gráfico da curva de aquisição, verificam-se produções corretas desde os 3:0 anos de idade. Dessa forma, analisar a curva de aquisição é fundamental, pois algumas crianças começam a produzir corretamente o segmento antes que outras até que, em determinada idade, a grande maioria precisa produzir adequadamente o segmento.

A aquisição acontece de maneira gradual, conforme visualizado na curva até que a criança atinge o domínio, no qual é observado um percentual de produção correta igual ou maior de 90% de produção correta, com nenhuma ou mínima alteração/erro na produção. Por exemplo, uma criança de 5:0 anos que na avaliação fonológica verificou-se que teve 65% de produção correta para o segmento /ɾ/, ao olhar no gráfico observou-se que esse percentual está próximo ao que é esperado aos 3;6 de idade (já sendo um indicativo de atraso ou alerta no progresso da aquisição) e que aos 5;0 esse segmento deveria ter um percentual de produção correta maior de 90%, indicando o domínio do fonema. Assim, a curva de aquisição permite verificar de maneira mais dinâmica se uma criança está dentro do esperado para a sua idade ou inferir atrasos ou alertas para atraso na aquisição fonológica.

Em relação a coda, os segmentos /N/ e /L/ foram adquiridos e dominados antes dos 3:0, /S/ aos 3:6 e /ɾ/ aos 4:6. Esses achados sugerem uma aquisição um pouco mais tardia do que referido por estudo^(9)^ que identificou uma ordem de aquisição semelhante, porém em idade mais precoce para os segmentos /S/ e /ɾ/ (/S/ entre 2:6 e 3:0 e /ɾ/ aos 3:10).

Dessa forma, o presente estudo confirmou que os segmentos do PB são adquiridos na seguinte ordem: plosivas e nasais, seguidas de fricativas e, por fim, líquidas/*tap*. Esses achados corroboram os estudos prévios de aquisição de segmentos do PB^(3,10,24)^.

A análise do *Onset* Complexo mostrou que aquisição e domínio ocorrem de maneiras distintas entre as diferentes possibilidades (Fricativa+/ɾ/, Fricativa+/l/, Plosiva+/ɾ/, Plosiva+/l/) de realização. As estruturas compostas por Fricativa+/ɾ/ e Plosiva+/ɾ/ foram adquiridas primeiro, aos 5:0, e o domínio foi aos 6:0. Por sua vez, as estruturas compostas por Plosiva ou Fricativa+/l/ foram adquiridas mais tarde, a Plosiva+/l/ foi adquirida aos 5:6 e o domínio aos 6:6 e a Fricativa+/l/ foi adquirida aos 6:0 e o domínio aos 6:6. Um estudo^(10)^ sugere que a estrutura de *onset* complexo pode ser adquirida entre 4:0 e 4:6. Outros estudos^(15,26)^ refere que os grupos constituídos por /ɾ/ são adquiridos antes dos formados por /l/, isso corrobora os achados deste estudo, bem como o domínio da estrutura, com idades próximas. Um pouco divergentes, os resultados do estudo^(10)^ que verificou que a estrutura composta pelo /l/ foi adquirida aos 4:0 e a estrutura com o /ɾ/ aos 5:0, mostrando que a estrutura composta pelo /l/ foi adquirida antes.

Devido as divergências referidas ao longo do estudo, torna-se necessário e importante realizar mais estudos sobre a aquisição fonológica do PB. Dessa forma, espera-se que esse trabalho com análises de aquisição fonológica de crianças típicas contribua de forma teórica e prática, permitindo identificar possíveis alterações durante essa cronologia e até mesmo na identificação de crianças com aquisição fonológica atípica^(1)^. Para o fonoaudiólogo, isso é fundamental, pois permite avaliar, intervir e tratar as crianças com aquisição fonológica atípica antes que alcancem o período destinado à alfabetização. As crianças atípicas parecem ser mais imaturas no que se refere a aquisição motora, especialmente na produção dos sons da fala se comparadas às crianças típicas^(27)^. Ainda, sugere-se que estudos futuros sobre aquisição fonológica sejam realizados com amostras maiores para que resultados possam ser generalizados para a população brasileira com suas diferentes características.

O formato de curva, para representar a aquisição fonológica, condiz com uma ferramenta que auxilia na dinâmica para entender o processo de aquisição fonológica, pois a criança parte do 0% até chegar aos 90% de produção correta ou mais quando alcança uma determinada idade. No formato de curva, a fácil visualização dos percentuais de produção correta nas diferentes faixas etárias, auxilia no comparativo entre as próprias crianças, com faixas semelhantes, aferindo o que seria esperado para sua idade.

É importante saber os parâmetros como a idade de aquisição e domínio dos fonemas para estabelecer se há ou não um atraso na aquisição, mas também é igualmente importante analisar essa progressão. Por exemplo, uma criança de 4:6 anos que na avaliação fonológica verificou-se que teve 65% de produção correta para o segmento /ɾ/. Se apenas os parâmetros forem observados, pode-se concluir que a criança está na idade de aquisição desse segmento, e que já começou a realizar corretamente, mas corre-se o risco de concluir que não há um atraso na aquisição desse segmento. Porém, ao olhar na curva de aquisição observa-se que o percentual de produção de 65% está próximo ao que é esperado aos 3:6 de idade (podendo ser um sinal de alerta para um atraso na aquisição desse segmento em relação aos seus pares). Dessa forma, analisar a curva de aquisição permite ao clínico confirmar se uma criança está dentro do esperado para a sua idade ou inferir atrasos na aquisição fonológica.

Estudos^(5,28)^ referem que na prática clínica dados normativos de aquisição podem ajudar os fonoaudiólogos a diferenciar crianças com desenvolvimento típico ou com atraso no desenvolvimento. Acredita-se que o fonoaudiólogo deva considerar além dos parâmetros de aquisição e domínio, o percentual de produção correta da curva para auxiliar no diagnóstico e terapia, propondo ações fonoaudiológica que auxilie na aquisição desse segmento.

Os achados deste estudo devem ser interpretados considerando algumas limitações, tais como: limitações demográficas da amostra, a qual foi recrutada apenas na região sul do Brasil e não terem sido avaliadas crianças menores de 3:0, pois alguns segmentos apresentam idades de aquisição e domínio anteriores.

## CONCLUSÃO

Neste artigo, foi analisada a aquisição fonológica dos segmentos do PB em *onset* simples, complexo e coda a partir da curva de aquisição. Os resultados mostram que, em *onset* simples, aos 3:0 estavam adquiridas e dominadas as plosivas, nasais e as fricativas /f/, /v/, /s/ e /z/. A aquisição de /ʃ/ e /l/ foi aos 3:0, /ʒ/ e /x/ aos 3:6, /ʎ/ aos 4:0, /ɾ/ aos 4:6. Porém, o domínio desses segmentos foi: /ʃ/ e /ʒ/ aos 4:0, /l/ e /x/ aos 3:6, /ɾ/ aos 4:6 e /ʎ/ aos 6:0. Em coda, a aquisição e o domínio de /N/ e /L/ foram antes dos 3:0, /S/ foi adquirido aos 3:6 e o domínio foi aos 4:0 e /ɾ/ aos 4:6 (mesma idade de aquisição e domínio). Em *onset* complexo, as estruturas compostas por Fricativa+/ɾ/ e Plosiva+/ɾ/ foram adquiridas aos 5:0 e o domínio aos 6:0, a Plosiva+/l/ foi adquirida aos 5:6 e o domínio aos 6:6 e a Fricativa+/l/ foi adquirida aos 6:0 e o domínio aos 6:6.

Ressalta-se que a aquisição e o domínio para algumas crianças podem ocorrer antes dessas idades encontradas, sendo importante analisar a curva de aquisição. Isso tem implicações significativas durante a avaliação e o diagnóstico de crianças com suspeitas de alterações dos sons da fala.

Além disso, observa-se que, à medida que as crianças vão se tornando mais velhas, a média de produção correta dos segmentos aumenta, possibilitando o incremento de novos segmentos em seu inventário fonológico.
